# Evaluation of Communicable Disease Surveillance System at Primary Health Care Centers in Jeddah, Saudi Arabia

**DOI:** 10.7759/cureus.19798

**Published:** 2021-11-22

**Authors:** Mohammed H Alshehri, Abdullah A Alsabaani, Amal H Alghamdi, Ruba A Alshehri

**Affiliations:** 1 Saudi Board of Preventive Medicine Program, Public Health Administration, Ministry of Health, Jeddah, SAU; 2 Department of Family and Community Medicine, College of Medicine, King Khalid University, Abha, SAU; 3 Primary Health Care Centers, King Abdulaziz Hospital, Jeddah, SAU

**Keywords:** saudi arabia, jeddah, communicable disease control, evaluation of performance, disease surveillance, primary health care centers

## Abstract

Introduction

Jeddah is one of the busiest and multicultural cities in Saudi Arabia. It poses a higher risk of importing and spreading emerging communicable diseases because of the increased international traffic during the seasons of Hajj and Umrah. The Saudi Ministry of Health (MOH) emphasizes the role of primary health care centers (PHCCs) as the first gate of the health care system. Therefore, having an efficient and effective communicable disease surveillance system (CDSS) at the level of PHCCs is crucial to provide early warning and sustain health security.

Methods

This study took place at all PHCCs in Jeddah city between September 2017 and January 2018 as a descriptive cross-sectional study. Data were collected from CDSS key informants using an interview-based questionnaire to evaluate the performance of CDSS by assessing its core and support functions at PHCCs.

Results

The majority (93%) of PHCCs had reporting forms, and all of them had working laboratories. However, about 41% of PHCCs had the standard manual and only in the Arabic language, 12% were performing basic data analysis, and none of them had a written plan for epidemic response. Although Internet access was available at only 33% of PHCCs, other resources such as computers, printers, and personal protective equipment (PPE) were available at all PHCCs.

Conclusion

CDSS at PHCCs had an acceptable performance especially in functions such as reporting, confirmation, and supervision. However, other functions such as detection, registration, data analysis, epidemic preparedness, and feedback need to be strengthened. More comprehensive evaluations are required to further enhance the CDSS in Jeddah and Saudi Arabia.

## Introduction

Emerging and reemerging communicable diseases have shown that we are continuously susceptible to health risks. The strengthening of national, regional, and global communicable disease surveillance systems (CDSSs) to provide early warning and sustain health security has been the main expert recommendation for the past two decades [1,2].

In the 1980s, the Saudi Ministry of Health (MOH) activated and developed preventive health services by adopting the primary health care (PHC) approach as one of its major health strategies. One of the key elements of this approach was preventing and controlling communicable diseases [3].

Recently, the Saudi Ministry of Health announced the launch of the first health system initiative of national health transformation named “New Model of Care” [4]. This model emphasizes the importance of preventive activities over curative ones and highlights the role of primary health care centers (PHCCs) as the first gate to service clients of the health care system.

Communicable diseases such as the Middle East respiratory syndrome coronavirus (MERS-CoV), dengue fever, and Alkhurma have a relatively higher incidence in Saudi Arabia and an alarming raised concern globally [5-7]. As one of the busiest and multicultural cities in Saudi Arabia, Jeddah has a population of about four million, with nearly half comprising foreigners [8]. In 2017 alone, the number of international passengers at King Abdulaziz International Airport exceeded 21 million [9]. The possibility of importation of an emerging communicable disease is highly significant, especially with increased population traffic during the seasons of Hajj and Umrah [10]. Therefore, as the first line of action for controlling this serious problem of emerging diseases, the implementation of an efficient and effective CDSS is crucial in order to have an immediate identification system of these diseases in place [11,12].

The evaluation of CDSSs should be periodic and comprise an assessment of core and support functions. Core functions include detection, registration, and confirmation of cases, reporting or notification, data analysis and interpretation, epidemic preparedness and response, and feedback and dissemination of the findings to stakeholders. Support functions, which are in place to facilitate the implementation of the core functions, include training, supervision, communication, and prioritizing resources [13,14].

To improve the capacity of CDSS to prevent and control communicable diseases, it is crucial to evaluate its performance at the level of PHC. This study evaluated the performance of CDSS by assessing its core and support functions at PHCCs in Jeddah, Saudi Arabia.

## Materials and methods

This study was conducted as a descriptive cross-sectional study at PHCCs in Jeddah city between September 2017 and January 2018. The study population comprised all PHCCs in Jeddah city (n = 45). From each PHCC, a key informant of CDSS, who must be a physician representing the head of CDSS at that PHCC, was selected.

The study tool was an interview-based questionnaire to assess the performance of CDSS at the level of PHCCs by interviewing the CDSS key informant at each PHCC and observing the indicators. It was originally developed by the WHO based on a conceptual framework for surveillance that categorizes the framework into eight core and four support functions measured with indicators to standardize assessments of CDSS [15]. Core functions included detection, registration, confirmation, reporting, analyses, epidemic preparedness epidemic response, and feedback. Support functions included supervision, training, communication, and resources.

Three out of 45 PHCCs (n = 3) were randomly selected for a pilot study to test the feasibility and validity and reliability of the tool. Accordingly, the tool was deliberately adapted with an acceptable level of reliability (Cronbach's α = 0.76) to be specific for the CDSS at PHCCs in Jeddah. The three PHCCs from the pilot study were excluded afterward. All the remaining PHCCs (n = 42) in Jeddah city were included in this study.

Data were analyzed using Stata Statistical Software (Release 14, StataCorp (2015), College Station, TX, USA). Frequency tables were computed to describe the variables. Categorical variables were summarized showing frequency and percentage of each category.

## Results

A total of 42 PHCCs were investigated. The key informants of CDSS at PHCCs were interviewed and provided their feedback on the performance of CDSS at the level of their PHCCs.

Core functions

Table 1 provides an overview of the CDSS core function performance at PHCCs.

**Table 1:** Assessment of the CDSS core functions at PHCCs

Function Indicator	PHCCs (n = 42)
Case Detection and Registration	
Availability of standard case definitions (SCDs)	17 (40.5%)
Availability of clinical register	14 (33.3%)
Complete clinical register	8 (19%)
Case Confirmation	
Availability of working laboratory	42 (100%)
Ability to collect blood, urine, and stool	42 (100%)
Capacity to handle specimens until transport	42 (100%)
Capacity to transport specimens to a higher laboratory	0 (0%)
Data Reporting	
Availability of reporting forms	39 (92.9%)
Submission of weekly reports in the last three months	7 (17%)
Submission of monthly reports in the last three months	7 (17%)
Data Analysis	
Availability of demographic data (population)	2 (4.8%)
Capacity to do data analysis (person, time, and place)	5 (11.9%)
Epidemic Preparedness and Response	
Manual for standard case management	17 (40.5%)
Prevention and control measures based on local data	3 (7.1%)
Feedback	
Received feedback reports from a higher level	6 (14.3%)

Case Detection and Registration

Standard case definitions (SCDs) were available in 17 PHCCs (41%) in the electronic format of “Guidelines for Surveillance and Control of Infectious Diseases,” which is published by Saudi MOH in Arabic language only. Although there was a daily register of cases at every clinic of all PHCCs, only 14 (33%) PHCCs had a dedicated clinical register for reported communicable diseases. Those registers were complete in eight (19%) of the 14 PHCCs.

Case Confirmation

All 42 PHCCs (100%) had working laboratories with the capacity to collect and handle blood, urine, and stool specimens until transportation. None of the 42 PHCCs (0%) had the capacity to transport or confirm identified diseases. Specimens were collected every week by a higher reference laboratory for confirmation.

Data Reporting

The reporting forms were available at the majority of PHCCs (n = 39, 93%) in an electronic format and had been accessible on demand during the last six months. When reporting diseases, forms were printed, filled manually, and sent via email (n = 18, 43%) or mobile applications such as WhatsApp (n = 24, 57%) without a formal mechanism to document them. Only seven (17%) PHCCs had submitted required weekly and monthly reports on time during the last three months.

*Data Analysis* 

Demographic data were available at two (5%) PHCCs. Analysis by means of person, time, and place was performed at only five (12%) PHCCs. The majority of PHCCs cited lack of trained staff and time as the main reason for not performing analysis regularly.

Epidemic Preparedness and Response

Seventeen (41%) PHCCs had a manual for standard case management. Three (7%) PHCCs reported the use of local data for prevention and control measures. However, none of the 42 (0%) PHCCs had a written plan for response in case of an outbreak.

Feedback

Feedback from higher levels was provided at six (14%) PHCCs.

Support Functions

Table 2 shows an overview of the CDSS support function performance at PHCCs.

**Table 2:** Assessment of the CDSS support functions

Function Indicator	PHCCs (n = 42)
Standards and Guidelines	
Availability of surveillance manual in English	0 (0%)
Availability of surveillance manual in Arabic	17 (40.5%)
Supervision and Training	
Supervisory visits in the last six months	34 (81%)
Training on CDSS in the last six months	24 (57.1%)
Resources	
Vehicle	0 (0%)
Computer	42 (100%)
Printer	42 (100%)
Statistical software	0 (0%)
Telephone service	42 (100%)
Internet access	14 (33.3%)
Projector	11 (26.2%)
Disinfectants and personal protective equipment (PPE)	42 (100%)

Standards and Guidelines

The surveillance manual published by Saudi MOH was up to date and easy to understand but available only in the Arabic language. The manual was found only in a digital format in 17 PHCCs (40%).

Supervision and Training

There were supervisory visits from a higher level at 34 (81%) PHCCs during the last six months ranging from one to three visits. Training on CDSS was provided for at least one physician or medical director at 24 (57%) PHCCs during the last six months.

Resources

Although all 42 (100%) PHCCs were provided with at least one computer and printer, none of the 42 (0%) PHCCs had statistical software. Despite the availability of telephone service in all 42 (100%) PHCCs, Internet access was only available in 14 (33%) PHCCs. A projector was available in 11 (26%) PHCCs. Availability of vehicles was a significant issue where none of the 42 (0%) PHCCs had a vehicle. Disinfectants and personal protective equipment (PPE) were available in all 42 (100%) PHCCs.

Satisfaction

Among the 42 key informants, 27 (66%) were not satisfied with the current CDSS. They commented that addressing issues such as not using an electronic system (90%), shortage of staff (81%), and insufficient training (28%) would improve the CDSS at PHCCs.

## Discussion

Jeddah is the main port for pilgrims who arrive from around 184 countries in the world to Makkah for Hajj or Umrah, making it one of the largest multicultural mass gatherings in the world [16]. Such a mass gathering has always been challenging for the Saudi government. The Saudi MOH has years of experience in dealing with this situation and tackling the danger of communicable diseases. However, there is always a potential risk of importing communicable diseases and spreading them locally and internationally [17]. Efforts are ongoing globally to evaluate such public health risks, and regular evaluation of CDSS is a necessity.

The CDSS in Saudi Arabia is well established and improving. However, most of the previous evaluations had either concentrated on attributes of timeliness and completeness or focused on specific diseases and national programs. Unfortunately, Saudi Arabia’s CDSS was seldom evaluated completely. In this study, we tried to describe and evaluate the CDSS in Jeddah at the level of PHCCs, which represent the peripheral level of the health system and the first gate that provides health care services to local communities.

In this study, the manual for standard case definitions was not available in 59% of PHCCs, which increases the possibility of poor case detection. The lack of knowledge of physicians about the manual existing or unavailability of Internet access could explain the low rate of case detection compared with a previous study in Jeddah [12]. Hopefully, continuous supervisory visits, which were frequently conducted (80%), may improve this situation.

The current manual [18] was updated and easily accessible. It was published in Arabic language only, which is the primary language of the country; however, all physicians had received their medical education in the English language. The digital version of the manual lacked an outline and had problems in text encoding that made it not searchable and challenging to navigate.

Although there were daily registers for cases at every clinic of all PHCCs, only 33% of PHCCs had a dedicated clinical register for reported communicable diseases, and only half of those registers were found complete. Because of absent, incomplete, or incorrect filling of disease or personal data, the usefulness of reporting is diminished as was found with an earlier study conducted in Jeddah to assess the reporting of communicable diseases [11].

Almost all PHCCs had well-functioning laboratories, where they could collect and handle specimens until transport. However, they had no capacity to confirm them, but specimens were collected weekly by a reference laboratory for confirmation. In comparison with a previous study conducted in Jeddah [12], our study showed an improvement in the capacity to collect specimens. Other studies from India and Sudan [19,20] reported weak laboratory infrastructure and logistics.

Forms were available in the majority (93%) of PHCCs in a digital format that could be printed and used, which shows an improvement from what was found in a previous study in Jeddah [12]. However, problems such as manual data reporting and using WhatsApp at the level of PHCCs, which leads to reduced data accuracy and the absence of a formal mechanism of documentation, weaken the system. Unfortunately, electronic data collection had not been implemented at PHCCs and was only required at higher levels. Weekly and monthly reporting were unsatisfactory, and that could be attributed to physicians’ lack of understanding of the significance of timeliness in reporting, absence of an electronic system, or shortage of staff with overwhelming daily tasks.

Limited data analysis at the PHC level in Jeddah indicates a shortage of qualified staff or a centralized system, and that may lead to collecting data without scientific interpretation. Similarly, other countries reported the same situation, including Sudan [20] and South Africa [21]. In contrast, a study for CDSS in Armenia [22] reported the use of descriptive statistics for data analyses at all levels of their health system.

The lack of a denominator for data analysis indicates that PHCCs had no idea about the actual magnitude of the communicable diseases in their covered area. This undesirably affects the use of surveillance data to do the recommended actions in time, and it may also affect the early detection of epidemics. Additionally, the majority of PHCCs did not have an epidemic threshold for common diseases, which may hinder the proper and early action on epidemics. This may be attributed to limited access to census data at the PHCCs and data analyses at higher levels without reporting the results back to PHCCs.

Epidemic preparedness and response at the level of PHCCs were insufficient. About 41% of PHCCs had a guideline to refer to standard case definitions and procedures. However, none of PHCCs had a written plan for epidemic management, which reduces the effectiveness of the structured response to outbreaks. Similar problems were also found in a study conducted in Sudan [20]. In contrast, another study in India [19] showed a relatively better preparedness and response to outbreaks.

Feedback is an essential element of the surveillance cycle [23]. What was found in this study indicates the absence of feedback in the majority of PHCCs, especially feedback from a higher level of the surveillance system, which may cause an incomplete loop of information. The lack or irregularity of feedback was also found in other studies from China, India, and Sudan [19,20,24]. The lack of motivating and clear feedback from the higher health administrations may weaken the CDSS performance and quality at PHCCs. Unfortunately, the current guidelines and regulations accentuate the need to provide feedback [18] but do not obligate health administrations to provide necessary feedback.

Supervision from higher levels was satisfactory and showed an improvement from what was found in a previous study done in Jeddah [12]. Routine supervisory visits provide training besides quality checks. However, with the lack of feedback, it may be a waste of resources within CDSS and a cause for hindering the achievement of the system goals.

Data analysis, feedback, and supervision are significantly affected by training [25]. The findings of this study showed that 54% of PHCCs had at least one physician who received training on CDSS in the last six months. Considering the short period and comparative studies, we could say that this finding was satisfactory.

The availability of sustainable resources is at the root of surveillance performance. In this study, PHCCs were well equipped with computers, printers, telephones, and faxes. However, access to the Internet was not available in two-thirds of PHCCs, which may act as a barrier in front of implementing or using an electronic solution. The lack of transport vehicles was a weakness where the health providers are expected to do active surveillance and investigation on a regular basis. Similar findings were reported from CDSS assessments in India [19]. Overall, the availability of resources is better than what was found in the 2009 study [12].

Limitations of the study

This study was limited to the PHC level only, which is a part of several levels in the Saudi health system. Other peripheral levels and private and military sectors were not included; however, they have different structures and policies that may affect their findings.

Although the questionnaire used for the assessment of CDSS performance was designed based on the conceptual framework of surveillance system recommended by the CDC and WHO, we did some modifications to make it more appropriate for the study setting, that is, to learn how the current CDSS performed at the PHC level in Jeddah. Therefore, it may be inapplicable to directly compare the findings described in this study with the results of similar studies from other countries due to the different study settings and data sources.

## Conclusions

Based on the findings, CDSS at the level of PHC in Jeddah had an acceptable performance especially in functions such as reporting, confirmation, and supervision. However, other functions such as detection, registration, data analysis, epidemic preparedness, and feedback need to be strengthened.

To improve the CDSS at PHCCs in Jeddah city, we recommend having multilingual and accessible guidelines for CDSS. Additionally, using an electronic system for managing CDSS at the level of PHCCs is a necessity for data accuracy and improving other attributes such as timeliness and simplicity. Shortage of qualified staff and overwhelming tasks need to be addressed by hiring new specialized employees or assigning existing employees to CDSS with proper training and incentives. Furthermore, feedback from higher levels is essential to complete the information loop and encourage PHCCs to improve their performance.

More detailed and thorough descriptions and evaluations are required to further enhance the CDSS not only in Jeddah city but also at the regional and national levels.
